# Towards Understanding the Microepidemiology of Lymphatic Filariasis at the Community Level in Ghana

**DOI:** 10.3390/tropicalmed9050107

**Published:** 2024-05-07

**Authors:** Jeffrey Gabriel Sumboh, Nii A. Laryea, Joseph Otchere, Collins S. Ahorlu, Dziedzom K. de Souza

**Affiliations:** 1 Department of Parasitology, Noguchi Memorial Institute for Medical Research, College of Health Sciences, University of Ghana, Accra P.O. Box LG 581, Ghana; jsumboh@noguchi.ug.edu.gh (J.G.S.); jotchere@noguchi.ug.edu.gh (J.O.); 2 Department of Epidemiology, Noguchi Memorial Institute for Medical Research, College of Health Sciences, University of Ghana, Accra P.O. Box LG 581, Ghana; nlaryea@noguchi.ug.edu.gh (N.A.L.); cahorlu@noguchi.ug.edu.gh (C.S.A.)

**Keywords:** microepidemiology, Lymphatic filariasis, spatial mapping, mass drug administration

## Abstract

Studies on the distribution of lymphatic filariasis (LF) have mostly focused on reporting prevalence at the community level and distribution at the district levels. Understanding the distribution patterns at community levels may help in designing surveillance strategies. This study aimed to characterize the spatial distribution of LF infections in four hotspot communities in Ghana. The research, involving 252 participants, collected demographic data, mass drug administration (MDA) information, household GPS coordinates, and antigen detection test results. The LF prevalence varied significantly among the communities, with Asemda having the highest (33.33%) and Mempeasem having the lowest (4.44%). Females had lower odds of infection than males (OR = 2.67, *p* = 0.003 CI: 1.39–5.13). Spatial analysis using kernel density, Anselin Local Moran’s, Getis-Ord Gi models, Ordinary Least Squares, and Geographic Weighted Regression revealed mixed patterns of spatial autocorrelation. This study identified LF hotspots, indicating clusters of high or low prevalence with some areas showing disparities between MDA coverage and LF positivity rates. Despite these hotspots, the overall distribution of LF appeared random, suggesting the importance of purposeful sampling in surveillance activities. These findings contribute valuable insights into the micro-epidemiology of LF, emphasizing the need for community-specific investigations to understand the factors influencing the effectiveness of MDA programs in controlling filarial infections. The study highlights the importance of refining surveillance strategies based on community-level distribution patterns.

## 1. Introduction

Lymphatic filariasis (LF), also known as elephantiasis, is a parasitic disease that affects millions of people in tropical and subtropical regions of the world [1,2]. It is caused through the transmission of microscopic worms called *Wuchereria bancrofti*, which are spread through the bites of infected mosquitoes [3]. The disease eventually leads to severe and disfiguring swelling of the limbs, genitalia, and breasts, and a variety of other health problems if not detected and treated early [4]. LF is a major public health concern, with an estimated 120 million people affected worldwide and over 1.3 billion people at risk of infection [5].

The World Health Organization (WHO) set a roadmap in 2021 aimed at eliminating LF by 2030 as a public health problem [6,7] and has brought together diverse groups including public–private partners and national governments to support the Global Programme to Eliminate Lymphatic Filariasis (GPELF) using two methods; mass drug administration (MDA) administered annually to entire communities in endemic regions and morbidity management and disability prevention for people with chronic complications [3,8,9].

MDA is the key strategy for the control and elimination of LF. MDA involves the administration of a single-dose combination of anti-parasitic drugs, such as diethylcarbamazine (DEC), ivermectin, and albendazole, to entire at-risk populations, regardless of whether they are infected [10]. The goal of MDA is to reduce the transmission of the disease by targeting the microfilariae that circulate in the blood [11]. By reducing the number of parasites in the population, the risk of transmission decreases, and the disease burden can be significantly reduced. The success of MDA programs relies on high coverage rates, which require strong community engagement and mobilization efforts. Community leaders, health workers, and volunteers play a critical role in educating people about the benefits of MDA and addressing any concerns or misconceptions they may have [12].

MDA has been highly effective in reducing the prevalence of LF in many areas. As of 2021, over 7 billion treatments have been delivered globally, and over 17 countries have been declared free of LF [13]. However, not all areas have seen the same level of success with MDA. The sustained, high-coverage MDA necessary to reach and maintain elimination targets can be quite challenging to achieve and, in some settings, the transmission of LF persists, leading to substantial suffering and disability [14,15]. Importantly, while MDA programs succeed in reaching their national and district-level coverage targets, focal transmission may persist at the sub-district or community levels and is often hard to detect with current M&E tools. Geospatial methods play a crucial role in identifying focal areas of disease transmission [16,17,18].

Geospatial maps provide a visual representation of the patterns showing the distribution of LF within communities with other relevant attribute data such as prevalence rates and potential breeding sites [9]. It gives a better understanding by providing a visual representation of the patterns of transmission, identifying high-risk areas, and helping programs plan targeted interventions [19].

Ghana was one of the early countries to adopt a national LF elimination strategy and has been undertaking MDA since 2001 [10,20]. At the time of writing this paper, 108/114 endemic districts have achieved success and stopped MDA and moved on to post-MDA surveillance; however, six districts have still not passed the Transmission Assessment Survey (TAS), despite 15–18 rounds of MDA. The inability of MDA to bring LF infection in these districts below the threshold where it is safe to stop mass treatment underscores the critical need to understand the local factors perpetuating LF transmission.

Micro-epidemiological investigations can shed light on local risk factors, such as unique social and cultural practices that may influence both exposure to the vector and treatment uptake, population mobility that could cause reintroduction of infection, and environmental factors that are more conducive to persistent LF transmission. By unraveling the intricacies of LF transmission at the micro level, tailored interventions can be developed to address the specific challenges hindering the achievement of TAS passing criteria, ultimately advancing progress towards LF elimination goals.

Spatial mapping represents an important starting point to visualize the patterns of LF infection at a micro level and to start to identify the risk factors likely to give rise to the observed infections. Importantly, spatial mapping can help the Ghana LF program identify high-risk areas in which to deliver targeted interventions.

## 2. Materials and Methods

### 2.1. Study Sites

**Figure 1:**
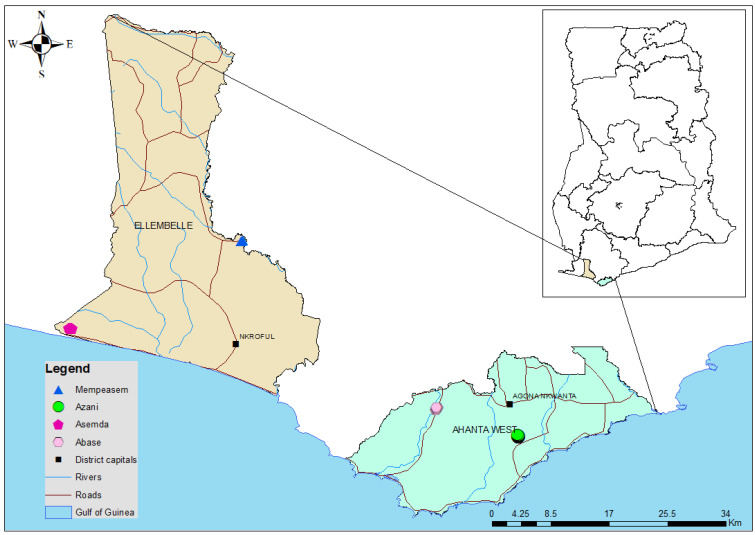
Map of Ghana showing the 4 communities in the two study districts. Base layers from (https://www.diva-gis.org/Data (accessed on 28 April 2023)).

The study was carried out in four rural communities in Ghana with a high incidence of lymphatic filariasis (LF) [21]. Asemda and Mempeasem are located in the Ellembelle district, while Azani and Abase are located in the Ahanta district of the western region of Ghana (Figure 1). These districts are in the moist evergreen ecological zone and receive rainfall throughout the year [22]. Farming and fishing are the main occupations in these communities and the dominant languages spoken are Ahanta and Nzema. The selected communities are all small. While the Ahanta West district passed TAS in 2022, MDA was still ongoing in Ellembelle at the time of this study in March 2023. The Ahanta West and Ellembelle districts have received 19 and 18 rounds of MDA, respectively.

### 2.2. Study Design

A community-wide assessment of LF was undertaken among community members 18 years and older. Each community was visited over a 3-to-4-day period, with sampling lasting from 6 am to 12 noon and from 3 pm to 6 pm in order to reach as many consenting adults in the community as possible. Due to the nature of this study, there was a mobilization and consenting team, a lab team, and a GPS team. Public announcements were made to inform the community of the presence of the study team at the central point. Door-to-door visits were also performed to sensitize community members to take part in this study. The following inclusion and exclusion criteria were followed.

Inclusion Criteria

1. Adults 18 years of age and above
2. Participants who have provided informed consent.
3. Participants who are residents of communities being studied.
4. Individuals who agree to blood collection and testing.

Exclusion Criteria

1. Children under 18 years.
2. Adults who refused consenting.
3. Adults who refuse blood collection and testing.
4. Adults who are sick or unwell at the time of the sampling.

At the central point written informed consent was obtained from all participants. Their demographic information and a simple questionnaire (see Supplementary File S1) on the knowledge attitude perception (KAP), participation in MDA, and drug acceptability was administered, after which day time blood collection and testing were conducted with the filarial test strip (FTS) [23]. Participants who tested positive by the FTS were asked to return at night (from 9 pm) for the collection of another blood sample to determine the presence and count of microfilaria by microscopy. After the FTS, the participants were accompanied to their respective houses and their household GPS coordinates were recorded using i-got GT-120 devices for spatial analysis. These data were used to map the distribution of the participants and their infection status within the communities. Attempts were made by the mobilization team over the 3-to 4-day period to reach all adults in the community.

### 2.3. Data Analysis

Stata version 17 and ArcGIS 10.8 were used for data processing and analysis. The demographic data were analyzed using descriptive statistics, including frequencies, percentages, and proportions, to provide insights into the distribution within communities and districts, with significance determined using chi-square tests. Logistic regression analysis was also performed to assess the significance of demographic characteristics and previous participation in mass drug administration. Ordinary least squares (OLS) and geographical weighted regression (GWR) were used to explore the community spatial variability with dependent factors such as age, while individual community-based spatial maps were created using a range of techniques, including kernel density and Anselin Local Moran I. In addition, the Getis-Ord Gi statistic model was utilized to identify spatial variations in prevalence and infection status [24,25].

### 2.4. Spatial Mapping and Predictive Risk Maps

The GPS coordinates of all the participants’ households were plotted within their respective communities. In cases where a compound had multiple individuals and positivity, graduated circles are used to indicate the numbers in the maps generated to provide a clear representation.

Kernel density was used for spatial analysis, which involved estimating the probability density function of the LF prevalence to reveal the underlying positivity distribution within the communities [26]. This is achieved by applying a “kernel” or smooth function to each data point in the dataset and combining them to obtain a density estimate. The kernel function is typically a bell-shaped curve, resembling a normal distribution, and its width determines the degree of emphasis given to neighboring households. The resulting density estimate is valuable for identifying prevalence patterns and predicting the areas where future infections may likely occur based on current prevalence. The formula used to calculate kernel density is:

$$\begin{array}{c} Density=\frac{1}{{\left(radius\right)}^{2}}\sum_{i=1}^{n}\left[\frac{3}{\pi }\cdot pop_{i}{\left(1-{\left(\frac{dist_{i}}{radius}\right)}^{2}\right)}^{2}\right]\\ For\;\;\;{dist}_{i}<radius \end{array}$$

where *i* = 1, …, *n* are the input points; $pop_{i}$ is the population field value of point one; and $dist_{i}$ is the distance between compounds and the (x, y) location.

### 2.5. Spatial Autocorrelation Analysis

The Anselin Local Moran’s analysis method was further used to determine the similarity of household prevalence to nearby households. This method was employed to recognize the spatial clusters and anomalies in the data collected. It measures the degree to which household prevalences’ in close proximity to each other exhibit similarity [27]. It is particularly useful in identifying clusters of high or low LF prevalence. The technique involves computing a local Moran’s I statistic for each observation in the dataset, which quantifies how closely related the prevalences are. By examining the local Moran’s I statistic, we detected clusters of similar observations (high–high) or dissimilar observations (low–high or high–low). The formula used to calculate the local Moran’s I statistic is:

$$I_{I}=\frac{Z_{i}-\bar{Z}}{\sigma^{2}}\sum_{j=1\cdot j\neq i}^{n}\left[W_{ij}(Z_{j}-\bar{Z})\right]$$

In the formula, $Z_{i}$ represents the value of the variable *Z* at location *i*, $\bar{Z}$ is the average value of *Z* in the sample of *n*, $Z_{j}$ denotes the value of the variable *Z* at all other locations (where *j* ≠ *i*), $\sigma^{2}$ is the variance of the variable *Z*, and $W_{ij}$ is a weight assigned based on the distance $d_{ij}$ between locations *i* and *j*. The weight $W_{ij}$ can be calculated as the inverse of the distance $d_{ij}$ or can be determined using a distance band, where samples within the distance band are given equal weight, while those outside the distance band are assigned a weight of 0.

The Getis-Ord Gi model further determined the difference between observed and expected household prevalences. It identified spatial clusters of high or low values. It is an extension of the local Moran’s I statistic, which measures the spatial autocorrelation and extent to which high or low values cluster together in a spatial dataset [28]. Getis-Ord Gi does this by comparing the values of each observation or data point to the values of its neighboring observations and then computing a z-score that indicates whether the observation is part of a cluster of high or low values. The z-score is obtained by dividing the difference between the observed value and the mean value of the neighboring observations by the standard deviation of the values of the neighboring observations. A positive Getis-Ord Gi statistic indicates that an observation has a high value and is surrounded by other observations with high values, suggesting a spatial cluster of high values. A negative Getis-Ord Gi statistic indicates that an observation has a low value and is surrounded by other observations with low values, indicating a spatial cluster of low values. A value of zero signifies no spatial clustering. Getis-Ord Gi is especially useful in detecting hotspots and coldspots in a spatial dataset, which are areas with significantly high or low values, respectively, such as LF infection rates. In determining significant hotspots, a high Z-score denotes the hotspot, and a coldspot is denoted by a low Z-score. This model is calculated as

$$\begin{array}{l} G_{i}=\frac{\sum_{j=1}^{n}W_{i,j(d)}b_{j}-\bar{b}\sum_{j=1}^{n}W_{i,j}}{s\sqrt{\frac{n\sum_{j=1}^{n}W_{i,j}^{2}-{\left(\sum_{j=1}^{n}W_{i,j}\right)}^{2}}{n-1}}} \end{array}$$

where *n* = total prevalence; $W_{i,j(d)}$ = spatial weight vectors for all the values within the distance (*d*); *d* = the distance between *i*th and *j*th location; $W_{i,j}$ = weight of the individual prevalence ($W_{i,j}$ = 1); $b_{j}$ = neighboring *j*th value; *b* = the average of all the prevalence; and *S* = the standard deviation.

The resulting predictive data for lymphatic filariasis infections were then plotted as heat maps.

## 3. Results

### 3.1. Study Participants and Community Characteristics

During this study, 252 participants were recruited from the four communities. Among these, 49 (19.44%) tested positive for lymphatic filariasis, and 203 (80.56%) tested negative. Asemda had the highest proportion of positive cases (29/87; 33.33%), followed by Azani (15/74; 20.27%), Abase (3/46: 6.52%), and Mempeasem (2/45: 4.44%). There was a significant association between sex and LF status (χ^2^ = 8.05, *p* = 0.005), with males having a higher proportion of positive cases (27.88%) than females (13.51%) (Table 1).

The association between participation in previous MDAs and test results was statistically significant (χ^2^ = 1.2. *p* = 0.002) with individuals who participated in previous MDA being less likely to have the disease compared to those who did not. LF positivity in Asemda (χ^2^ = 5.9, *p* = 0.015) was statistically significant compared to the other communities. No significant association was observed between the infection and the different age groups (Table 2).

### 3.2. Associations between Demographic Variables, MDA, and Infection

In the multiple logistic regression analysis conducted, the various factors, specifically community, sex, age group, and participation in previous MDA in relation to FTS positivity had different correlations. The odds of getting infected within the different communities was found to be 0.82, but this was not statistically significant (*p* = 0.258 CI: 0.58–1.16), indicating no association between the community of residence and the likelihood of testing positive for LF. Similarly, no association was found with the age grouping (OR = 1.12; *p* = 0.231 CI: 0.93–1.35), and participation in previous MDA (OR = 2.39; *p* = 0.072 CI: 0.92–6.18). However, a significant association was observed with sex. Females had a lower odd of infection than males (OR = 2.67; *p* = 0.003). Overall, the regression model accounted for a relatively small proportion (6%) of the variability in the FTS outcome, as indicated by the pseudo R-squared value of 0.0607, suggesting that other factors not included in the model may influence an individual’s likelihood of testing positive for LF.

### 3.3. Hotspots for Community Infections

**Figure 2:**
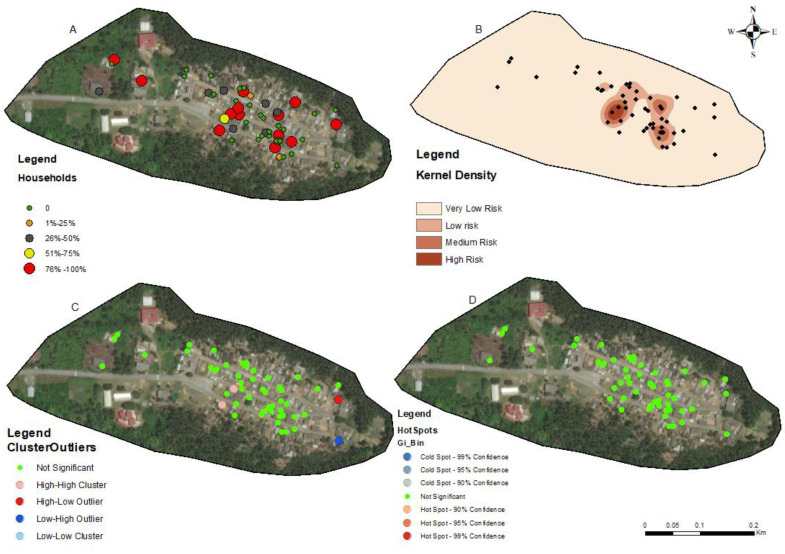
Spatial analysis of data from Asemda community, Ellembelle district. (**A**) Distribution of FTS positivity by household, (**B**) Kernel density-based spatial prediction of the likelihood of lymphatic filariasis infection; (**C**) hotspots for cluster and outlier LF positive distribution (Anselin Local Moran’s); (**D**) Spatial distribution of hotspots and coldspots (Getis-Ord Gi). Base layers from (https://www.diva-gis.org/Data (accessed on 17 May 2023)).

**Figure 3:**
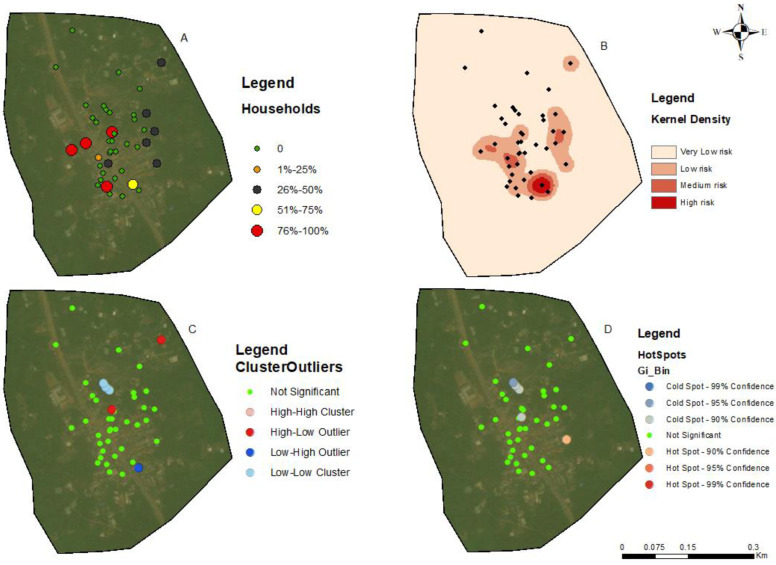
Spatial analysis of data from Azani community, Ahanta West district. (**A**) Distribution of FTS positivity by household; (**B**) Kernel density-based spatial prediction of the likelihood of Lymphatic Filariasis infection; (**C**) Hotspots for cluster and outlier LF positive distribution (Anselin Local Moran’s); (**D**) Spatial distribution of hotspots and coldspots (Getis-Ord Gi). Base layers from (https://www.diva-gis.org/Data (accessed on 17 May 2023)).

**Figure 4:**
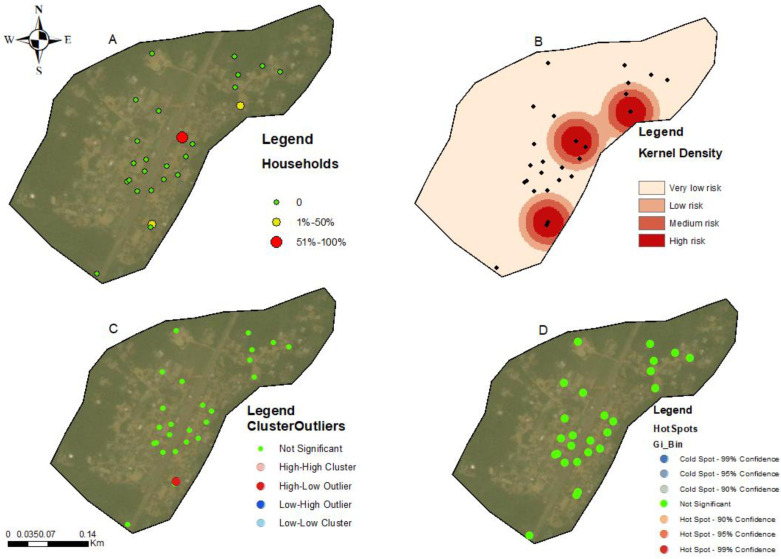
Spatial analysis of data from Abase community, Ahanta west district. (**A**) Distribution of FTS positivity by household; (**B**) Kernel density-based spatial prediction of the likelihood of lymphatic filariasis infection; (**C**) hotspots for cluster and outlier LF positive distribution (Anselin Local Moran’s); (**D**) Spatial distribution of hotspots and coldspots (Getis-Ord Gi). Base layers from (https://www.diva-gis.org/Data (accessed on 17 May 2023)).

**Figure 5:**
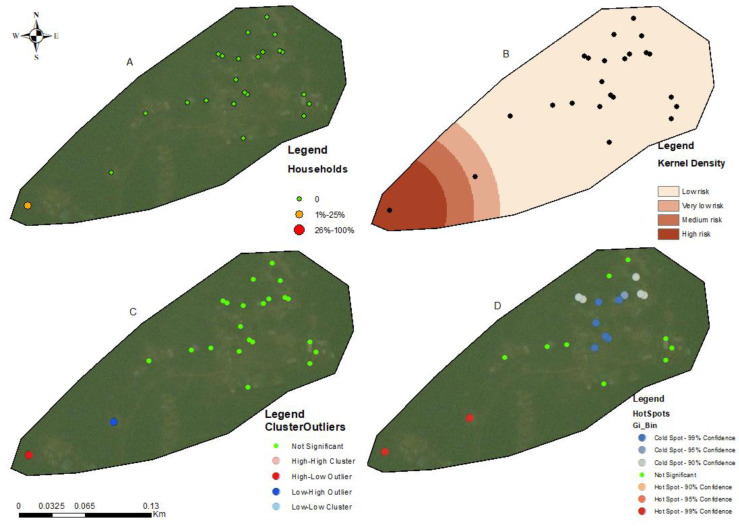
Spatial analysis of data from Mempeasem community, Ellembelle district. (**A**) Distribution of FTS positivity by household; (**B**) Kernel density-based spatial prediction of the likelihood of lymphatic filariasis infection; (**C**) hotspots for cluster and outlier LF positive distribution (Anselin Local Moran’s); (**D**) spatial distribution of hotspots and coldspots (Getis-Ord Gi). Base layers from (https://www.diva-gis.org/Data (accessed on 17 May 2023)).

The households in all four communities exhibited a proximity to each other in terms of spatial distribution. The number of people tested for LF per household within these communities ranged from 1 to 5 individuals (Figure 2A, Figure 3A, Figure 4A, and Figure 5A). In the output maps, higher values corresponded to areas where more individuals within households underwent LF testing, whereas lower values indicated areas with a lower number of individuals tested. This suggests that the positivity rate would have likely gone up if more people from households willingly consented to test. At the community level, the testing participation rate was Abase: 4.56%, (46/1008), Asemda: 5.00% (87/1741), Azani: 6.61% (74/1119) and Mempeasem: 5.73% (45/784). Considering that Ghana has an estimated adult population of around 65% (18 and above), Abase, Asemda, Azani, and Mempeasem had adult testing rates as 7.02% (46/655), 7.69% (87/1131), 10.18% (74/727), 8.82% (45/510), respectively.

The kernel density distribution analysis was performed using the positive cases of LF per household to estimate the spatial distribution of infections (Figure 2B, Figure 3B, Figure 4B, and Figure 5B). The resulting maps illustrate the smoothed density of LF-positive cases across the study communities. Higher-density areas correspond to areas with a concentrated number of positive cases, indicating potential hotspots of increased LF infection prevalence. This distribution highlights the spatial patterns and clusters of LF positivity.

Anselin’s local Moran’s hotspot analysis was also conducted using the positivity rates for LF to identify statistically significant clusters and outliers. The analysis reveals specific areas on the map that exhibit significant spatial clusters of high or low LF positivity (Figure 2C, Figure 3C, Figure 4C, and Figure 5C). Infectivity clusters were mostly found around the outskirts of the communities due to a combination of factors, mosquito reservoirs, community population distribution, etc.

The Getis-Ord Gi hotspot analysis was conducted using the positivity rates for lymphatic filariasis (LF) to identify statistically significant hotspots within the study communities (Figure 2D, Figure 3D, Figure 4D, and Figure 5D). This analysis pinpointed specific areas on the map that exhibited significant clusters of high or low LF positivity. High-value clusters indicated areas with a dense concentration of LF-positive cases, indicating areas of heightened infection prevalence. Conversely, low-value clusters represented areas with a lower number of LF-positive cases. The Getis-Ord Gi analysis provided valuable insights into the spatial patterns of LF, enabling the identification of hotspots and areas that may require targeted interventions for LF control and prevention.

### 3.4. Spatial Autocorrelation

The Moran’s index indicated the degree of spatial association between neighboring households (Table 3). Asemda showed a slight positive spatial autocorrelation (Moran’s Index: 0.007), suggesting neighboring households with similar LF positivity levels. In contrast, Abase (−0.085) and Azani (−0.019) exhibited negative spatial autocorrelation, indicating a dispersion pattern where neighboring households have contrasting LF positivity rates. Mempeasem (0.043) displayed a positive Moran’s index, suggesting a clustering pattern of LF positivity in neighboring households. The *p*-values for Asemda, Abase, and Azani (0.816, 0.642, and 0.942, respectively) were above the significance threshold (*p* > 0.05), suggesting that the observed spatial autocorrelation in LF positivity in these communities could be due to random chance rather than true spatial patterns. For Mempeasem, the *p*-value (0.057) was marginally close to the significance threshold, indicating a possible clustering pattern, as indicated by the model.

The general G statistic assessed the global spatial autocorrelation. The communities exhibited non-significant values. Asemda (*p* = 0.815), Abase (*p* = 0.578) and Azani (*p* = 0.309) showed no significant spatial clustering or dispersion in LF positivity (Figure 2D, Figure 3D, Figure 4D, and Figure 5D). Mempeasem had undefined data, making interpretation impossible.

**Figure 6:**
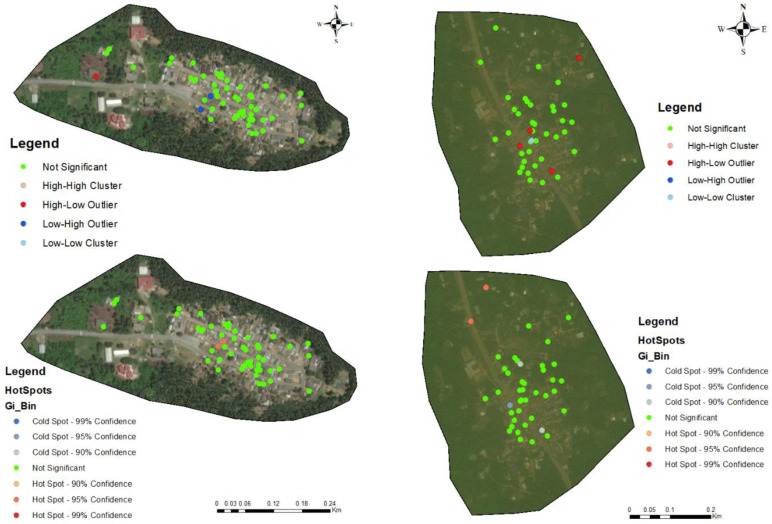

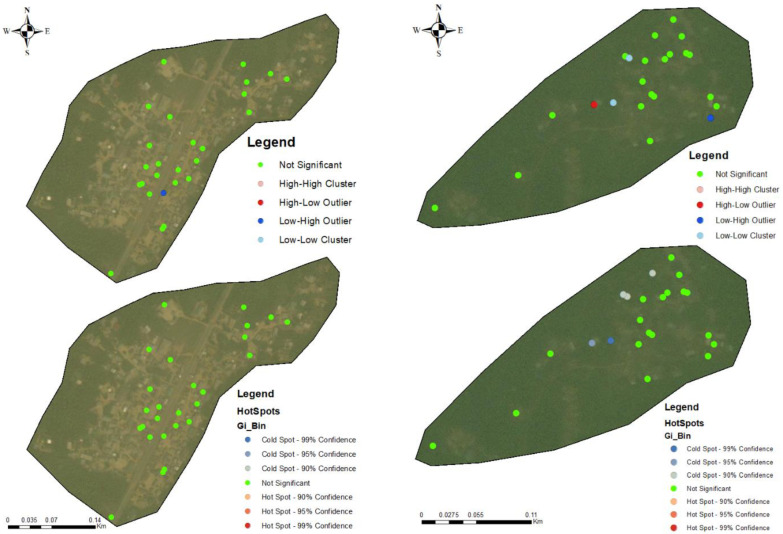
Spatial distribution for cluster and outlier (Anselin Local Moran’s), and hotspots and coldspots (Getis-Ord Gi) on self-reported MDA across the communities. Base layers from (https://www.diva-gis.org/Data (accessed on 17 May 2023)).

The spatial analysis of participant engagement on self-reported coverage in the last MDA treatment round within households in each community showed a negative Moran’s index across the communities (Table 4). These values suggest a weak negative spatial autocorrelation indicating that individual households with higher self-reported coverage are not clustered together in proximity, and the same holds true for those with lower coverage, which are less clustered than what would be expected by chance and may consistently report higher or lower coverage compared within each community (Figure 6). Also, people within a household exhibited similar treatment coverage. This spatial heterogeneity further reveals that participant engagement varies across different areas within each community and within different households.

Furthermore, the z-scores for all communities show that the observed spatial autocorrelation is lower than what would be expected by chance while non-significant corresponding *p*-values imply that the household’s distribution within each community do not deviate significantly from random distribution.

The observed General G values were positive for all communities showing a tendency towards positive spatial autocorrelation (Table 3). This suggests that households with similar levels of participant engagement are somewhat clustered together within each community. The Expected General G values were relatively close to the observed values indicating that the actual spatial autocorrelation variability is not different from what would be expected by chance, showing a random spatial distribution of participant engagement across each community. The corresponding *p*-values shows that the spatial patterns of participant engagement are not statistically significant, except for Azani, where the *p*-value (0.02) indicates that the spatial pattern of participant engagement is statistically significant, deviating from random distribution.

### 3.5. Ordinary Least Squares (OLS) and Geographic Weighted Regression Analysis (GWR)

OLS regression analysis was conducted to investigate the relationship between age and LF positivity across the four communities: Abase, Asemda, Azani, and Mempeasem. Overall, the intercept values showed statistically significant associations with LF positivity across all communities (Abase: *p* < 0.001, Asemda: *p* < 0.001, Azani: *p* < 0.001, Mempeasem: *p* < 0.001). However, while the coefficient for age did not reach statistical significance in Abase, Azani, and Mempeasem (*p* > 0.05 for all), it was statistically significant in Asemda (*p* = 0.02).

These findings suggest that while there is a consistent baseline association between the intercept values and LF positivity across the communities, age alone may not be a significant predictor for positivity in most settings. However, in Asemda, age does appear to be a significant predictor, albeit with a relatively modest effect size.

GWR allows for the parameters of regression to vary spatially for continuous data. Using age as the determining factor again and only continuous variable in relation to LF prevalence, the values across the four communities—Asemda, Abase, Azani, and Mempeasem—provide valuable insights into LF infections with implications for understanding prevalence.

Asemda demonstrates a relatively small bandwidth of 0.041, signifying a localized effect within a limited spatial range. Abase and Mempeasem presented even smaller bandwidths of 0.00098 and 0.0012, respectively, emphasizing highly localized effects within extremely narrow distances. Azani, with a bandwidth of 0.0031 showcases a more balanced influence within a moderate spatial scale. In fitting the model for GWR, residual squares which represents the difference between the observed values and the values predicted by the model revealed community spatial variations. Abase stands out with lower residual squares (1.93), suggesting a more optimal fit of the model in capturing the variability in FTS positivity. In contrast, Asemda displayed higher residual squares (17.31), indicative of a less optimal fit. Azani falls in between with moderate residual squares (10.91), while Mempeasem has the lowest residual squares (0.78), reflecting the strongest fit comparatively. Mempeasem had a more consistent relationship between age and FTS positivity while Abase and Azani were moderate implying a balanced level of spatial variability. Asemda, suggested greater spatial heterogeneity in the relationship.

Examining R^2^ and adjusted R^2^ values determined the proportion of explained variances in community infectivity. Abase emerged with the highest R^2^ (0.30) and adjusted R^2^ (0.18), indicating the most substantial explanatory power. Mempeasem, while exhibiting a strong R^2^ (0.18), showed a lower adjusted R^2^ (0.09). Asemda and Azani present lower values, suggesting less explanatory power. These results underscore the importance of tailoring interpretations to the specific characteristics of each community, providing valuable insights for public health interventions and community-specific strategies.

The assessment of residuals from both OLS and GWR across the four communities evaluated model performance in capturing variability for LF positivity. With the OLS analysis, Abase exhibited lower residuals, suggesting a more optimal fit of the model in capturing LF positivity variability. Conversely, Asemda showed higher residuals, indicating a less optimal fit of the model. Azani’s residuals may reflect potential model inadequacy in explaining LF positivity within the community, while Mempeasem’s residuals reflected the overall fit of the model. The GWR analysis provided additional insights into spatial variations in model fit. Abase showed lower residual squares, indicating a stronger fit of the model and potential spatial homogeneity in LF positivity. A relatively small bandwidth in Asemda suggested localized effects of age on LF prevalence, highlighting spatial heterogeneity. Azani, with lower residual squares, potentially indicated a more optimal fit of the model and highlighted potential areas for underprediction or overprediction. Mempeasem exhibited lower residual squares, suggesting a stronger fit compared to other communities and potential areas with more consistent relationships between age and LF positivity.

### 3.6. FTS Positivity and Participation in Mass Drug Administration

**Figure 7:**
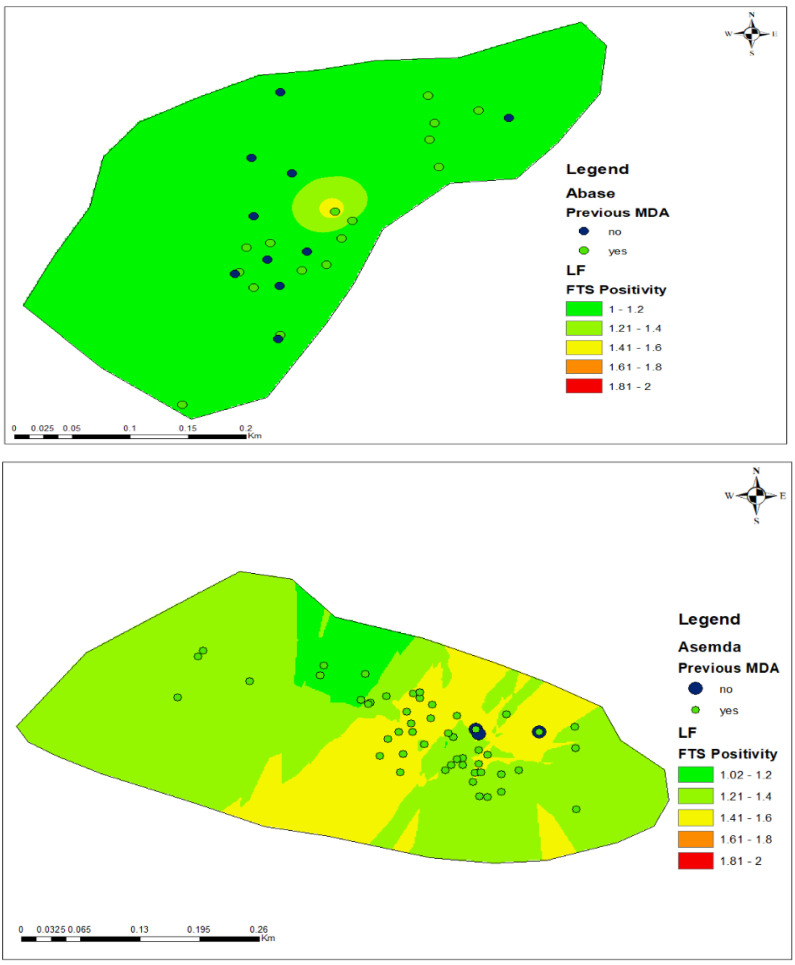

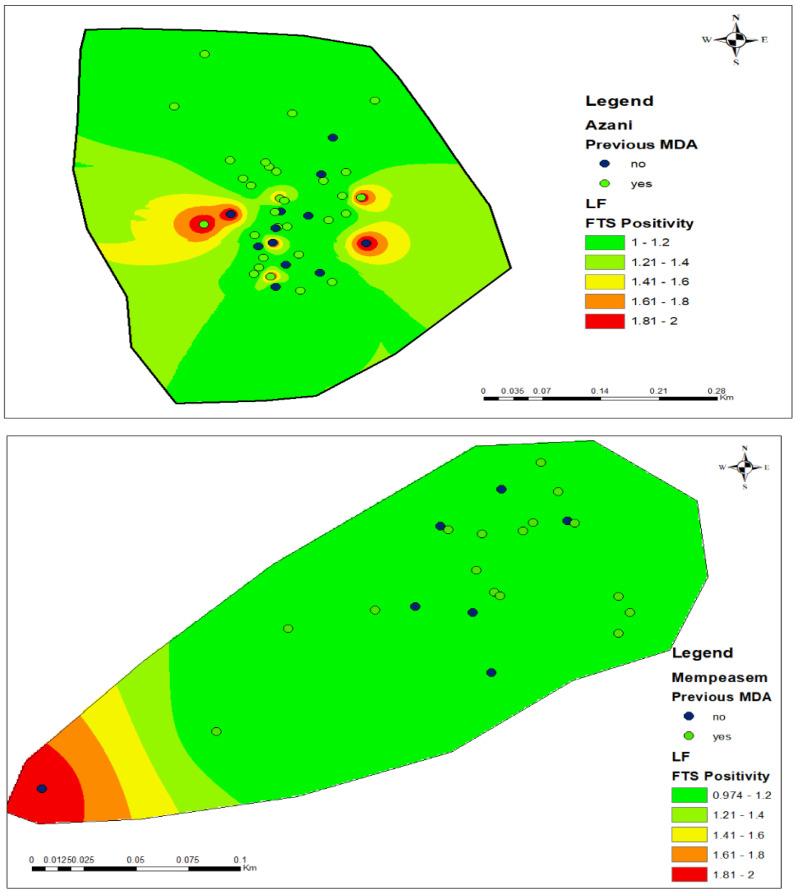
Distribution of predicted risk of LF using the inverse distance weighted model and adherence to previous MDA across the communities. FTS positivity is depicted as a percentage.

The comparison between FTS status (Table 1) and MDA coverage, based on participant self-reported coverage from the last treatment time-point in these communities, surveyed had Abase’s FTS positivity rate at 6.52%, while having a moderate MDA coverage of 71.7%. In contrast, Asemda had a considerably higher FTS positivity rate of 33.33%, but also exhibited a high MDA coverage of 97.7%. Azani fell in between with an FTS positivity rate of 20.27% and an MDA coverage of 75.7%, and Mempeasem had the lowest FTS positivity rate at 4.44% alongside an MDA coverage of 75.6%. Participants residing in households that did not participate in the most recent MDA showed a high likelihood of infection in Azani and Mempeasem (Figure 7).

## 4. Discussion

This study sought to systematically characterize the spatial distribution of lymphatic filariasis infections in four communities located in the Ahanta West and Ellembelle districts of Ghana to gain insights into the disease patterns, identify high-risk areas, and utilize this information for effective decision making.

The significant association between LF status and the study communities suggests variations in prevalence and transmission dynamics. Asemda emerged as a high-risk community with a substantial proportion of LF-positive cases. This highlights the need for targeted interventions such as intensified surveillance, vector control measures, and focused treatment campaigns in Asemda to reduce LF transmission and prevalence [29,30]. Participant engagement within households in the last MDA round shows spatial heterogeneity in the self-reported coverage within each community. The weak negative spatial autocorrelation and non-significant spatial patterns in the Moran’s index (Table 3) indicate that participant engagement is influenced by unique factors specific to different areas within each community. The variability in engagement levels highlights the need for targeted and community-specific approaches to enhance participant engagement. The General G statistic also showed the spatial autocorrelation within each community. While Asemda and Abase show a slightly higher observed spatial autocorrelation than expected, Azani exhibits a significant negative deviation from random distribution. Mempeasem displays a moderate observed spatial autocorrelation but lacks statistical significance (Table 3). Understanding these spatial patterns can inform targeted interventions, especially community members who do not adhere to MDA to enhance engagement and optimize MDA program effectiveness in these communities.

The observed significant association between LF status and sex suggests that males may have a greater likelihood of exposure compared to females, as confirmed by [31]. This highlights the importance of implementing gender-specific interventions and targeted awareness campaigns to address the specific risk factors and behaviors that contribute to the higher prevalence of LF among males. By focusing on gender-specific approaches, public health efforts can effectively tackle the underlying factors that contribute to the disparity in LF prevalence between males and females, ultimately leading to the more successful LF control and prevention strategies [32].

The utilization of kernel density analysis allows for the identification of high-density hotspots of lymphatic filariasis (LF) transmission, which may enable targeted interventions and resource allocation to be more effective. By deploying preventive measures such as focused vector control and mass drug administration campaigns in localized areas with the highest disease burden, the overall transmission of LF can be reduced [33]. The spatial autocorrelation analysis employed in this study, including Moran’s Index and General G statistic, provide insights into the clustering or randomness of LF infections. These findings help uncover the underlying spatial patterns of LF transmission, allowing for the identification of high-risk areas and informing targeted surveillance and control strategies, such as prioritizing engagement, testing, and treatment of community members residing in these identified high-risk hotspots. Such a targeted approach allows for a more focused allocation of resources and interventions, optimizing the impact of preventive measures and contributing to the overall control of LF. Moreover, spatial analysis aids in spatial prioritization, highlighting the localized nature of infections and emphasizing the need for tailored interventions in specific areas [34,35]. The hotspot analysis highlighted specific areas within the communities that showed the potential of the high concentration of positive cases due to transmission. In Asemda and Azani (Figure 2B and Figure 3B), the kernel density maps indicated the presence of infections within the central portions of these communities. It was observed that these central areas had a higher concentration of households with more members, which could potentially attract mosquitoes and contribute to the higher prevalence of infections [36,37,38]. In the case of Abase (Figure 4B), the density map revealed infections extending along the eastern side of the community. This area has a higher proportion of forested areas compared to other parts of the community. The presence of more forested areas might create suitable breeding grounds for mosquitoes, leading to a higher likelihood of LF transmission and subsequent infections in this specific area [39]. For Mempeasem (Figure 5B), the infections concentrated at the entrance of the community. This area had a lower density of households and was characterized by a higher proportion of forested areas. The combination of fewer households and more forested surroundings might contribute to a higher risk of LF transmission in this location [40]. It is also important to note that the sampling size does not necessarily reflect the assumption that areas with more participants will have a higher risk. In Figure 5A, for example, the area with the highest risk had less participants and was isolated from the rest of the community.

Upon comparing the degree of spatial clustering in each community and between households (ranges from −1 to +1), positive values indicated positive spatial autocorrelation, i.e., households with high (or low) LF positivity were surrounded by neighboring households with similarly high (or low) positivity. Negative values also indicated negative spatial autocorrelation, where high (or low) positive households were surrounded by neighboring households with negative values [41,42,43]. Abase and Azani (Table 2) exhibited negative values, indicating negative spatial autocorrelation. This suggests that households with high LF positivity in these communities are surrounded by households with lower LF positivity or vice versa. In contrast, Asemda and Mempeasem (Table 2) showed positive values, indicating positive spatial autocorrelation. This suggests the presence of clusters or spatial patterns of similar LF positivity values within these communities.

Similarly, statistically significant hotspots and coldspots for infections were measured from the Getis-Ord Gi. High positive community values showed that households with high LF positivity were surrounded by neighboring households with similarly high positivity, suggesting hotspots. Negative values indicated households with low LF positivity surrounded by neighboring areas with similarly low positivity, suggesting coldspots [44]. This model suggests that the presence of LF-positive households does not necessarily indicate a high risk of transmission to nearby households, and the absence of positive households does not guarantee a lack of risk in the surrounding area within these smaller communities studied. This highlights the complexity of LF transmission dynamics and the need to consider multiple factors beyond individual household status [27]. Factors such as mosquito breeding sites, vector behavior, and environmental conditions can influence the spread of LF within a community [4,45,46]. Asemda, Abase, and Azani (Table 2) showed positive values for the General G statistic, indicating significant hotspots of high LF positivity. This suggests the presence of concentrated areas with a high LF infection prevalence within these communities. However, Mempeasem had a NaN value, indicating that it did not meet the criteria for significant hotspots or coldspots based on the chosen threshold.

The application of OLS revealed associations between LF positivity and age, highlighting the importance of understanding broad-scale patterns of disease transmission. GWR analyzed the infection’s diverse spatial patterns and relationships between age and prevalence. The localized nature of LF transmission, as reflected in bandwidth and effective number values, underscores the need for community-specific interventions. The varying model fits, as indicated by residual squares highlighting the importance of tailoring public health strategies to the unique characteristics of communities [47]. As GWR allows for the consideration of spatial variability, this approach contributes to a more targeted and nuanced understanding of LF spread, ultimately aiding in the development of effective and localized interventions [48]. Both regression results highlight the relationships between LF positivity and key predictors, varied across the different communities. This localized approach revealed clusters of heightened LF transmission risk (Figure 7), suggesting the presence of hotspot areas where targeted interventions may be most effective.

The identification of hotspot areas through OLS and GWR analyses enables policymakers to prioritize resources and interventions in areas with the highest risk of LF transmission. Targeted measures, such as intensified MDA campaigns and vector control efforts, can be implemented to effectively reduce LF prevalence in these hotspots. Community-specific interventions tailored to the localized risk can include greater community engagement and participation. By involving local communities in LF control efforts, such as enhanced community health education programs and outreach activities, policymakers can aid the effectiveness and sustainability of control initiatives. The spatial insights provided by both OLS and GWR analyses underscore the importance of robust surveillance and monitoring systems for LF control. The continuous monitoring of LF prevalence and transmission dynamics at the local level is essential for the timely detection of changes in disease patterns and the effectiveness of control measures. Addressing the multifactorial nature of LF transmission requires collaboration across multiple sectors, including health, environment, and sanitation. Policy efforts should focus on fostering intersectoral collaboration to address the underlying determinants of LF transmission, such as access to vector control and improved healthcare.

Azani and Abase are located in the Ahanta West district that had passed TAS less than a year before this study, while Mempeasem and Asemda in Ellembelle are still undergoing MDA. In conducting epidemiological surveys, the random selection of participants and not necessarily the willingness of participants to be part of interventions is critical in determining the infection and transmission thresholds in communities. This includes extending assessments, both parasitological and entomological, to households on the outskirts as much as possible. Assessments by NTD programs are generally based on the willingness of the participants, with the assessors setting up in a central location and waiting for participants. While the type of assessment implemented in this study may not be easy to replicate in routine programmatic activities, the results are important in designing epidemiological assessments to determine the transmission of LF in districts either for monitoring and evaluations during MDA or surveillance after MDA. In Ghana, monitoring and evaluation surveys for LF by the NTD program are based on community members who self-report to a common location for FTS or microfilaria testing. This may not provide a representative sample of the community, especially if participants who take part in the MDA are the same as those who take part in these surveys.

The results of this study emphasize the need to enhance activities to reach individuals who do not participate in MDA, requiring a multi-faceted approach that combines education, community engagement and tailored communication to address specific concerns and misconceptions. By implementing these strategies, MDA programs can strive for higher coverage rates and move closer to the goal of LF elimination. The study can also be enhanced in the future by integrating other community behavioral characteristics to optimize the spatial autocorrelation methods. Both methods used require the careful consideration of community definitions and assumptions. Nevertheless, when interpreted with caution and in combination with other spatial analysis techniques, these methods can provide valuable insights into the spatial structure and relationships within LF incidence and distribution [49].

There are some limitations to this study. First, it was conducted in relatively small communities, thereby affecting the sample sizes and the possible interpretation of the results. During the study, the adults available in the community were reached, except those who refused participation. Unfortunately, data on non-participation were not collected to better understand this group, although studies in a neighboring district identified around 4% of individuals who refused all interventions [10]. The small sampling size and the FTS positivity rates may also impact the spatial autocorrelation analysis. Some community members also went about their normal economic activities and could not be reached during the 3 or 4 days sampling and the hours during which the team was in the communities. As such, the likelihoods of the sampling being biased cannot be overruled. A census of the communities was not taken prior to sampling, and as such, the profile of non-participants and number of households was not available for further analysis. It should be noted that the same situation does apply to NTD interventions where only those who are willing are assessed and non-participants are usually not accounted for. In this study, however, we went a step further to reach out to reluctant individuals and map the coordinates of all those who were tested. Furthermore, it is important to note that the spatial autocorrelation analysis used in this study primarily focused on the spatial patterns of LF infections and did not consider contextual factors such as socioeconomic conditions and environmental covariates that could potentially influence disease transmission dynamics [21,50]. While incorporating such contextual information into the analysis may enhance the practical relevance and accuracy of the findings [51], it is unlikely that environmental covariates could differ significantly between the communities and change the transmission of LF in these very small communities located in moist evergreen ecological zone that receives rainfall throughout the year [22]. Despite these limitations, the results of the mapping reveal that the distribution of infection across all four communities is random and does not follow a clear pattern.

## 5. Conclusions

In this study, we characterized the spatial distribution of LF positivity in four communities in the Ahanta West and Ellembelle districts of Ghana. Our findings suggest that household LF infection does not exhibit a significant degree of spatial clustering at the community level. Interestingly, there does not appear to be a clear relationship between self-reported MDA coverage and the community-wide prevalence of LF. Any future studies investigating community-level infection assessment should follow a random household selection to ensure a representative sample.

## Figures and Tables

**Table 1:** Frequency distribution and percentage of participants by community, district, sex, age, and previous mass drug administration (MDA) for lymphatic filariasis.

	Total	Positive FTS	Negative FTS		
	N = 252 (%)	N = 49 (%)	N = 203 (%)	Statistic	*p*-Value
Community				Chi = 22.11	<0.001
Azani	74 (29.37)	15 (20.27)	59 (79.73)		
Abase	46 (18.25)	3 (6.52)	43 (93.48)		
Asemda	87 (34.52)	29 (33.33)	58 (66.67)		
Mempeasem	45 (17.86)	2 (4.44)	43 (95.56)		
District				Chi^2^ = 2.88	0.089
Ahanta West	120 (47.62)	18 (15.00)	102 (85.00)		
Ellembelle	132 (52.38)	31 (23.48)	101 (76.52)		
Sex				Chi^2^ = 8.05	0.005
Female	148 (58.73)	20 (13.51)	128 (86.49)		
Male	104 (41.27)	29 (27.88)	75 (72.12)		
Age				Chi^2^ = 5.73	0.572
≤20	13 (5.16)	2 (15.38)	11 (84.62)		
21–30	75 (29.76)	12 (16.00)	63 (84.62)		
31–40	40 (15.87)	8 (20.00)	32 (80.00)		
41–50	49 (19.44)	8 (16.33)	41 (83.67)		
51–60	34 (13.49)	8 (23.53)	26 (76.47)		
61–70	29 (11.51)	6 (20.69)	23 (79.31)		
71–80	6 (2.38)	2 (33.33)	4 (66.67)		
80>	6 (2.38)	3 (50.00)	3 (50.00)		
Participation in previous MDA				Chi^2^ = 2.61	0.106
Yes	200 (79.37)	43 (21.50)	157 (78.50)		
No	52 (20.63)	6 (11.54)	46 (88.46)		

**Table 2:** Frequency distribution of community, participant age characteristic, and participation in the last MDA according to sex.

		N	Positive (%)	Negative (%)	Chi^2^	*p*-Value
Community						
Abase		46	3 (6.5)	43 (93.5)	1.22	0.27
	Males	17	2 (11.8)	15 (88.2)		
	Females	29	1 (3.5)	28 (96.6)		
Asemda		87	29 (33.3)	58 (66.7)	5.9	0.015
	Males	41	19 (46.3)	22 (53.7)		
	Females	46	10 (21.7)	36 (78.3)		
Azani		74	15 (20.3)	59 (79.7)	1.61	0.205
	Males	20	6 (30.0)	14 (70.0)		
	Females	54	9 (16.7)	45 (83.3)		
Mempeasem		45	2 (4.4)	43 (95.6)	1.53	0.216
	Males	26	2 (7.7)	24 (92.3)		
	Females	19	0 (0.0)	19 (100)		
Age						
≤20		13	2 (15.4)	11 (84.6)	2.03	0.155
	Males	7	2 (28.6)	5 (71.4)		
	Females	6	0 (0.0)	6 (100)		
21–30		75	12 (16.0)	63 (84.0)	3.53	0.06
	Males	26	7 (27.0)	19 (73.0)		
	Females	49	5 (10.2)	44 (89.8)		
31–40		40	8 (20.0)	32 (80.0)	3.63	0.057
	Males	18	6 (33.3)	12 (66.7)		
	Females	22	2 (9.1)	20 (90.9)		
41–50		49	8 (16.3)	41 (83.7)	0.04	0.84
	Males	20	3 (15.0)	17 (85.0)		
	Females	29	5 (17.2)	24 (82.8)		
51–60		34	8 (23.5)	26 (76.5)	0.15	0.702
	Males	15	4 (26.7)	11 (73.3)		
	Females	19	4 (21.1)	15 (78.9)		
61–70		29	6 (20.7)	23 (79.3)	2.65	0.103
	Males	11	4 (36.4)	7 (63.6)		
	Females	18	2 (11.1)	16 (88.9)		
71–80		6	2 (33.3)	4 (66.7)	1.5	0.221
	Males	2	0 (0.0)	2 (100)		
	Females	4	2 (50.0)	2 (50.0)		
80>		6	3 (50.0)	3 (50.0)	1.2	0.273
	Males	5	3 (60.0)	2 (40.0)		
	Females	1	0 (0.00)	1 (100)		
Participation in previous MDA						
Yes		200	43 (21.50)	157 (78.5)	10.07	0.002
	Males	79	26 (32.9)	53 (67.1)		
	Females	121	17 (14.1)	104 (85.9)		
No		52	6 (11.5)	46 (88.5)	0.01	0.92
	Males	25	3 (12.0)	22 (88.0)		
	Females	27	3 (11.1)	24 (88.9)		

**Table 3:** Parameters of spatial autocorrelation for LF positivity across the communities.

Spatial Parameters	Asemda	Abase	Azani	Mempeasem
Moran’s index:	0.007037	−0.084898	−0.019971	0.043047
Expected index:	−0.019608	−0.04	−0.02439	−0.045455
Variance:	0.002905	0.009348	0.003744	0.002159
z-score:	0.233218	−0.464386	0.072227	1.9049
*p*-value:	0.815592	0.642371	0.942421	0.056793
Observed general G	0.010687	0.003277	0.005307	nan
Expected general G	0.011232	0.010113	0.008678	0.019148
Variance:	0.000005	0.000151	0.000011	inf
z-score:	−0.233428	−0.555806	−1.017551	nan
*p*-value:	0.815429	0.578343	0.308891	nan

**Table 4:** Parameters of spatial autocorrelation for LF self-reported MDA coverage across the communities.

Spatial Parameters	Asemda	Abase	Azani	Mempeasem
Moran’s index:	−0.048013	−0.088814	−0.060009	−0.052879
Expected index:	−0.019608	−0.04	−0.02439	−0.045455
Variance:	0.002918	0.010323	0.004088	0.009874
z-score:	−0.525878	−0.480445	−0.557114	−0.074718
*p*-value:	0.598973	0.630911	0.577449	0.940439
Observed General G	0.011734	0.011612	0.005906	0.015728
Expected General G	0.011232	0.010113	0.008678	0.019148
Variance:	0.000001	0.000003	0.000002	0.000009
z-score:	0.417736	0.815016	−2.197311	−1.156848
*p*-value:	0.67614	0.415063	0.027998	0.247335

## Data Availability

The datasets analyzed during the current study are available from the corresponding author on reasonable request.

## Supplementary Materials

The following supporting information can be downloaded at: https://www.mdpi.com/article/10.3390/tropicalmed9050107/s1, Supplementary File S1: Intrument for data collection.
