# Supplementary material for: Towards Understanding the Microepidemiology of Lymphatic Filariasis at the Community Level in Ghana
Source: Trop Med Infect Dis. 2024 May 7;9(5):107. doi: 10.3390/tropicalmed9050107 (PMC11125695; doi:10.3390/tropicalmed9050107)
Supplement: Supplementary file 1 [file tropicalmed-09-00107-s001.zip › tropicalmed-2961112-supplementary.pdf]

Supplementary File S1

**Study Area**					
01	Region: Western	02	Interviewer:	03	Community:
04	Municipality:	05	Date of Interview		
	House number:				
	GPS Coordinates:				

	Question	Response
**Socio-Demographic characteristics**		
1	Name of participant	
2	Is name in the register?	Yes<br>No
3	Age (in completed years)	
4	Gender	Male<br>Female
5	Position in the family	Male head of household<br>Female head of household<br>Child
6	Level of Education	No formal education<br>Primary<br>JHS<br>SHS<br>Technical/Vocational<br>Tertiary<br>Other, specify_______________
7	Religious affiliation	Christian<br>Muslim<br>Traditional<br>No religion<br>Other, specify_________________
8	Main occupation	Farming<br>Fishing<br>Farming + Fishing<br>Trade/Business<br>Official employee<br>Casual labor<br>Other, specify_________________
9	Relationship status	Single<br>Married<br>Divorced/separated<br>In a relationship but not married<br>Remarried<br>Widowed
**Knowledge Of Lymphatic Filariasis (Elephantiasis)**		

10	Do you know or have you ever heard of LF?	Yes<br>No
11	If yes to Q10, how did you hear of this disease?	CDD<br>Disease control officer<br>Community health nurses<br>Media (TV, radio, newspapers, internet etc.)<br>Friends<br>Relatives<br>Other (specify):
12	If No to Q10, what means of communication will make you hear about the disease faster?	
13	Do you know what causes LF?	Yes<br>No
14	If yes to Q13, what do you think causes the disease?	Mosquitoes<br>Pollution<br>Cold Weather<br>Wind<br>Poor hygiene/Dirty environment<br>Sweet foods/Sugar<br>Oily foods/peanuts<br>Rain/Standing water<br>Eggs<br>Witchcraft. curse<br>Don't know<br>Other (specify):
15	Do you know any sign or symptom of LF?	Yes<br>No
16	If yes to Q14, which would you consider?	High body temperature (fever)<br>Swollen scrotum<br>Swollen breasts<br>Swollen legs<br>Sores on the body<br>Don't know<br>Others (specify):
17	Do you think you are at risk of being infected with LF	Yes<br>No
**Compliance To Treatment**		
18	Did you know about the LF MDA campaign for LF?	Yes<br>No

19	If yes to Q18, how did you hear about it?	Radio<br>TV<br>Gongong beater<br>Poster<br>Friend/social network<br>CDD<br>Other: ------------------------
20	Did you receive any drugs during the last treatment round?	Yes<br>No
21	If Yes to Q20, from where did you collect these drugs?	CDD came to my house<br>I went to CDD's House<br>From the community centre<br>N/A
22	If No to Q20, why not?	Absent<br>Underage<br>Pregnant/breastfeeding<br>Fear of sides effects<br>Not being informed<br>CDD didn't come to house<br>Don't believe in free things<br>I was away at work<br>We do not have a CDD<br>Tired of swallowing the drug<br>Drug is not effective<br>I think I am not having the disease<br>Don't like drugs<br>Travel<br>Other, Specify ________________
23	If yes to Q21, did you swallow the drug?	Yes<br>No<br>Not applicable (did not take drug)
24	In the last 3 MDAs (including this year), how many times have you swallowed the MDA drugs? (**Have the register with you and cross check the answers they give with what is in the register**).	**a) Response given**<br>Zero<br>Once<br>Twice<br>Three times<br><br>**b) Answer in register**<br>Zero<br>Once<br>Twice<br>Three times
25	How many other times (treatment rounds) have you previously taken the drugs?	Once<br>Twice<br>Three times<br>Four times<br>Five times<br>Six times

		More than 6 times<br>Forgotten<br>Other (specify):
**Social Networks**		
26	Do you know anyone who has refused/stopped taking these drugs?	Yes<br>No
27	If yes to Q26, how many people do you know?	
28	If yes to Q26, what were the reason given?	Fear of sides effects<br>Don't believe in free things<br>Tired of swallowing the drug<br>Drug is not effective<br>Not having the disease<br>Don't like drugs<br>Other, Specify ________
29	Do you know any CDD personally (is (s)he your friend)?	Yes<br>No
30	Are you aware how CDD's are recruited?	Yes (explain)<br>No
31	Can you mention by name up to 10 close friends you have in this community?	

LF Drug acceptability

	Please rate your opinion on the following statements:	Disagree a lot	Disagree	Agree	Agree a lot
32	These drugs work against LF				
33	These drugs work against itching				
34	These drugs work against intestinal worms				
35	I would take this treatment again				
36	I would recommend this treatment to my relatives				
37	I would be willing to change my family's routine so that we took the treatment again				
38	I liked this treatment				

30	This treatment is a good way to help our health problems here					
40	Overall, this treatment will help my community					
